# The therapeutic effects of adipose-derived mesenchymal stem cells on obesity and its associated diseases in diet-induced obese mice

**DOI:** 10.1038/s41598-021-85917-9

**Published:** 2021-03-18

**Authors:** Hala jaber, Khodr Issa, Ali Eid, Fatima A. Saleh

**Affiliations:** 1 Department of Nutrition and Dietetics, Faculty of Health Sciences, Beirut Arab University, Beirut, Lebanon; 2 Department of Molecular Diagnostics, Doctors’ Center Laboratories, Beirut, Lebanon; 3 Department of Pharmacology and Toxicology, Faculty of Medicine, American University of Beirut, Beirut, Lebanon; 4 Department of Basic Medical Sciences, College of Medicine, QU Health, Qatar University, Doha, Qatar; 5 Biomedical and Pharmaceutical Research Unit, QU Health, Qatar University, Doha, Qatar; 6 Department of Medical Laboratory Technology, Faculty of Health Sciences, Beirut Arab University, Beirut, 115020 Lebanon

**Keywords:** Mesenchymal stem cells, Endocrine system and metabolic diseases

## Abstract

Obesity is a global public health concern associated with increased risk of several comorbidities. Due to the limited effectiveness of current therapies, new treatment strategies are needed. Our aim was to examine the effect of adipose-derived mesenchymal stem cells (AD-MSCs) on obesity and its associated diseases in a diet-induced obese (DIO) animal model. C57BL6 mice were fed with either high fat diet (HFD) or CHOW diet for 15 weeks. Obese and lean mice were then subjected to two doses of AD-MSCs intraperitoneally. Mice body weight and composition; food intake; blood glucose levels; glycated hemoglobin (HbA1c), intraperitoneal glucose tolerance test and atherogenic index of plasma (AIP) were measured. Pro-inflammatory cytokines, tumor necrosis factor-α and interleukin-6, were also determined. AD**-**MSCs treatment reduced blood glucose levels, HbA1c and AIP as well as improved glucose tolerance in DIO mice. In addition, MSCs caused significant attenuation in the levels of inflammatory mediators in HFD-fed mice. Taken together, AD-MSCs were effective in treating obesity-associated diabetes in an animal model as well as protective against cardiovascular diseases as shown by AIP, which might be partly due to the attenuation of inflammatory mediators. Thus, AD-MSCs may offer a promising therapeutic potential in counteracting obesity-related diseases in patients.

## Introduction

Obesity is a global health problem, particularly in light of its association with increased risk of several comorbidities, all-cause mortality and impaired quality of life^1^. The prevalence of obesity is increasing worldwide in adolescents and adults of both genders and has doubled between 1980 and 2016^2^. It is also expected that by 2030, 57.8% of the population will be obese^3^. Moreover, obesity is associated with a spectrum of obesity-related morbidities resulting from complications affecting different aspects of physiology. These morbidities include hyperlipidaemia, cardiovascular diseases, and type 2 Diabetes^4^. Currently, a wide variety of interventions has been proposed for obesity and its related disorders in clinical settings^5,6^. However, effective therapies to cure obesity and its comorbidities are still lacking. Thus, investigators are in an urgent need to look for potential treatments that show effectiveness. Stem cell-based therapies currently hold great promise to treat various diseases including cardiovascular diseases (CVD); neurodegenerative diseases, muscular degenerative disorders; hematopoietic and immune system disorders; liver injuries; diabetes; arthritis; cancers as well as other diseases in our body that could take benefit of stem cell therapy^7,8^.

Particularly, Mesenchymal stem cells (MSCs) are an attractive candidate for a wide range of therapeutic applications^9^. Their relative ease of isolation from many tissues such as the bone marrow, adipose tissue and umbilical cord coupled with their multilineage differentiation capacity, have made them potential tools to treat many diseases^10^. In recent years, adipose-derived MSCs (AD-MSCs) are emerging due to their abundance, ease of collection, rapid expansion and high proliferation capacity^11^. Additionally, studies have confirmed that they can maintain the basic phenotype of MSCs over long-term culture up to Passage 10^11^. Stem cell therapy in obesity and its related diseases has been suggested by several animal studies but results seem to be unclear, making it far and difficult to move into clinical settings^12–14^. Therefore, well-designed studies are needed to determine efficacy in the animal model before proceeding to humans.

The purpose of this study was to investigate the effect of AD-MSCs on body weight, body composition, glycaemic control and dyslipidaemia in an in vivo animal model. An experimental model of obesity was established in mice under chronic high-fat diet (HFD) feeding for 15 weeks. The therapeutic potential of intraperitoneal AD-MSC infusion to treat dietary-induced obesity and its associated disorders such as diabetes and CVD in a mouse model of HFD–induced obesity was elucidated in this study. The mechanism by which AD-MSCs produce their therapeutic effects was also explored.

## Results

### Generation of HFD-induced obese and diabetic mice

**Figure 1:**
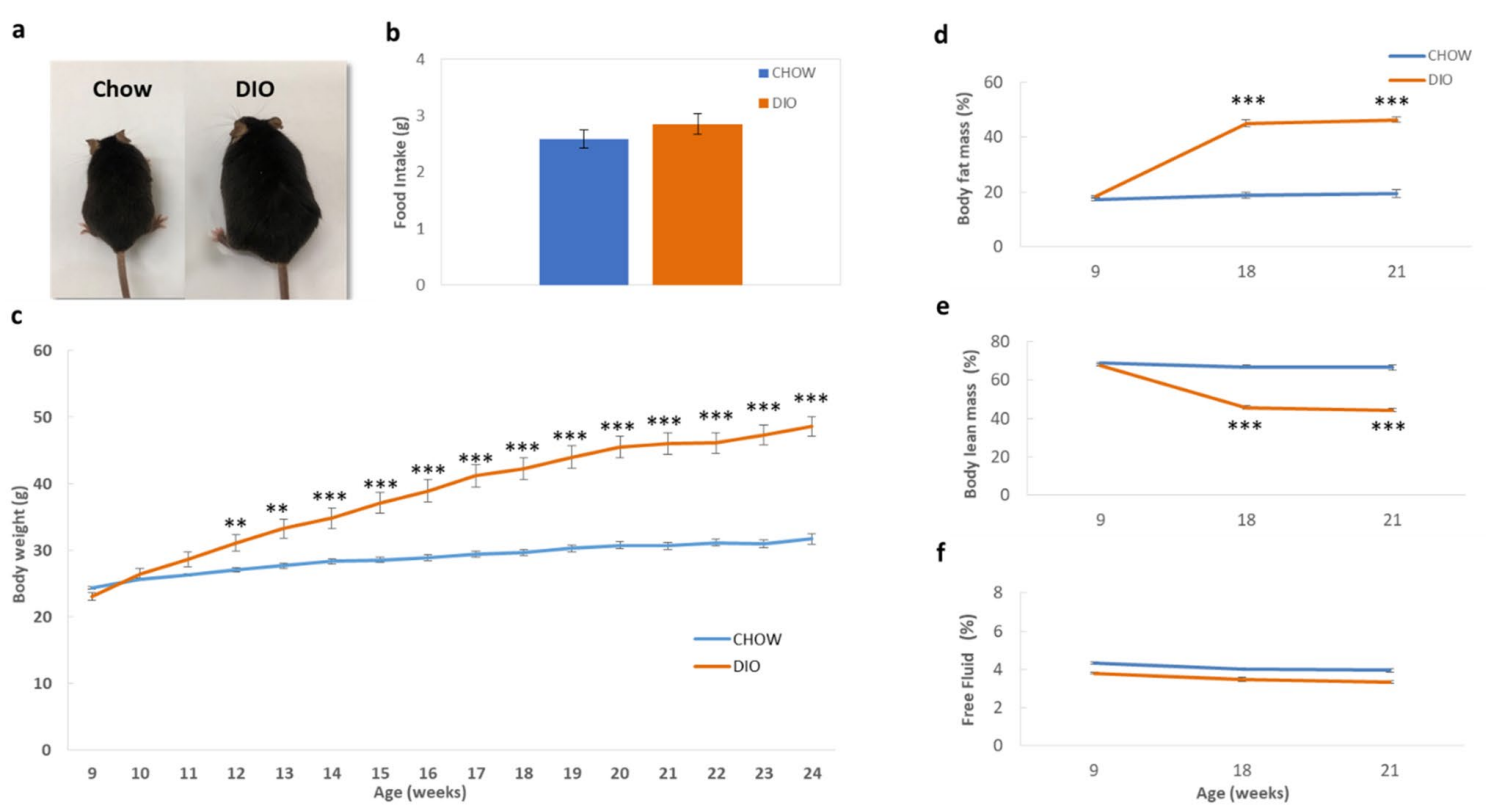
Generation of Diet-induced obese (DIO) mice by high-fat diet feeding for 15 weeks. (**a**) Representative images of DIO and CHOW mice (control). (**b**) Food intake of DIO and CHOW mice at 21 weeks of age. (**c**) Changes in body weights of DIO and CHOW mice undertaken weekly during the 15 week-feeding period. (**d**) Body-fat percentages, (**e**) body lean percentages and (**f**) free fluid percentages of DIO and CHOW mice at 9, 18 and 21 weeks of age. n = 12, **p < 0.01, ***p < 0.001.

8-week-old male C57BL/6 mice were allowed to adapt for 1 week to the new environment. Then, mice were fed for 15 weeks with either a high-fat diet (HFD) to generate diet-induced obese (DIO) mice or a standard CHOW diet to produce lean mice (Fig. 1a). Food intake was measured after 12 weeks of feeding. Nevertheless, there was no significant difference in food intake between DIO and CHOW mice (Fig. 1b). Regarding body weight, mice fed the HFD displayed significantly higher body weights than mice fed with the CHOW diet after only 3 weeks of feeding (*p* < 0.01). This increase in body weight in the HFD-fed mice continued throughout the 15-week feeding period to reach 48.63 ± 1.46 g in DIO mice as compared to 31.75 ± 0.73 g (*p* < 0.001) in CHOW mice at 24 weeks of age as shown in Fig. 1c. As presented in Fig. 1d–f, there were alterations in body composition parameters upon high fat dieting, as determined using Minispec LF110 body composition analyzer. At baseline, no significant differences in parameters were observed. However, after 9 weeks of HFD feeding, there was significant elevation (*p* < 0.001) in fat mass percentage in the HFD group (Fig. 1d). At week 21, DIO mice had 46.4% body fat mass as compared to 19.5% in CHOW mice (*p* < 0.001). On the other hand, lean mass percentage decreased significantly in DIO mice at weeks 18 and 21 (*p* < 0.001) (Fig. 1e). However, free fluid content was not significantly different between both mice groups at all time points (Fig. 1f). It can be concluded that DIO mice continued to gain weight over the time period of the study and that was mainly due to the increase in total body fat mass rather than lean mass.

**Figure 2:**
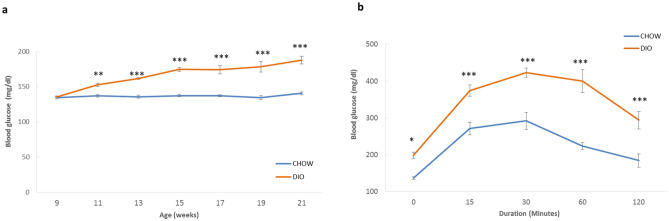
Blood glucose levels and Glucose tolerance in DIO and CHOW mice. (**a**) Changes in blood glucose levels of DIO and CHOW mice that were determined every 2 weeks during the 15 week-feeding period. (**b**) Intraperitoneal Glucose Tolerance Test (IPGTT) was performed at the end of the feeding period. n = 12, *p < 0.05, **p < 0.01, ***p < 0.001.

Blood glucose levels were also measured regularly every 2 weeks in both mice groups. After only 2 weeks on HFD, blood glucose significantly increased from 135.5 ± 1.33 mg/dl to 152.5 ± 2.25 mg/dl (*p* < 0.01) and remained elevated to reach 187.7 ± 5.6 mg/dl on week 21 as compared to 140.5 ± 1.99 mg/dl in CHOW-fed mice (*p* < 0.001) (Fig. 2a). However, blood glucose measurements showed no significant increase in the control group throughout the 15-week feeding period. This indicates that blood glucose levels were altered by the HFD thus inducing hyperglycemia. A two-hour intraperitoneal glucose tolerance test (IPGTT) was also performed after 15 weeks of diet feeding to assess glucose homeostasis in mice. After 6 h of fasting (t = 0 min), there was a significant increase (P < 0.05) in blood glucose levels in DIO mice (199 ± 8.4 mg/dl) versus control mice (136.6 ± 3.63 mg/dl) (Fig. 2b). Now in order to determine the body’s ability to metabolize glucose, a 2 g/kg of glucose bolus was injected intraperitoneally (IP). DIO mice showed a rise in glucose levels (374 ± 15.5 mg/dl) after 15 min of infusion with a peak occurring after 30 min (423 ± 12.6 mg/dl) (*p* < 0.001) (Fig. 2b). Two hours later, both groups showed a reduction in glucose levels with the DIO group displaying significantly *(p* < 0.001) higher blood glucose level as compared with the CHOW-fed mice with a mean of 294 ± 24 mg/dl and 184.3 ± 18.14 mg/dl, respectively. These data suggest that the HFD caused glucose intolerance in mice.

### Effect of AD-MSCs on body weight and composition

**Figure 3:**
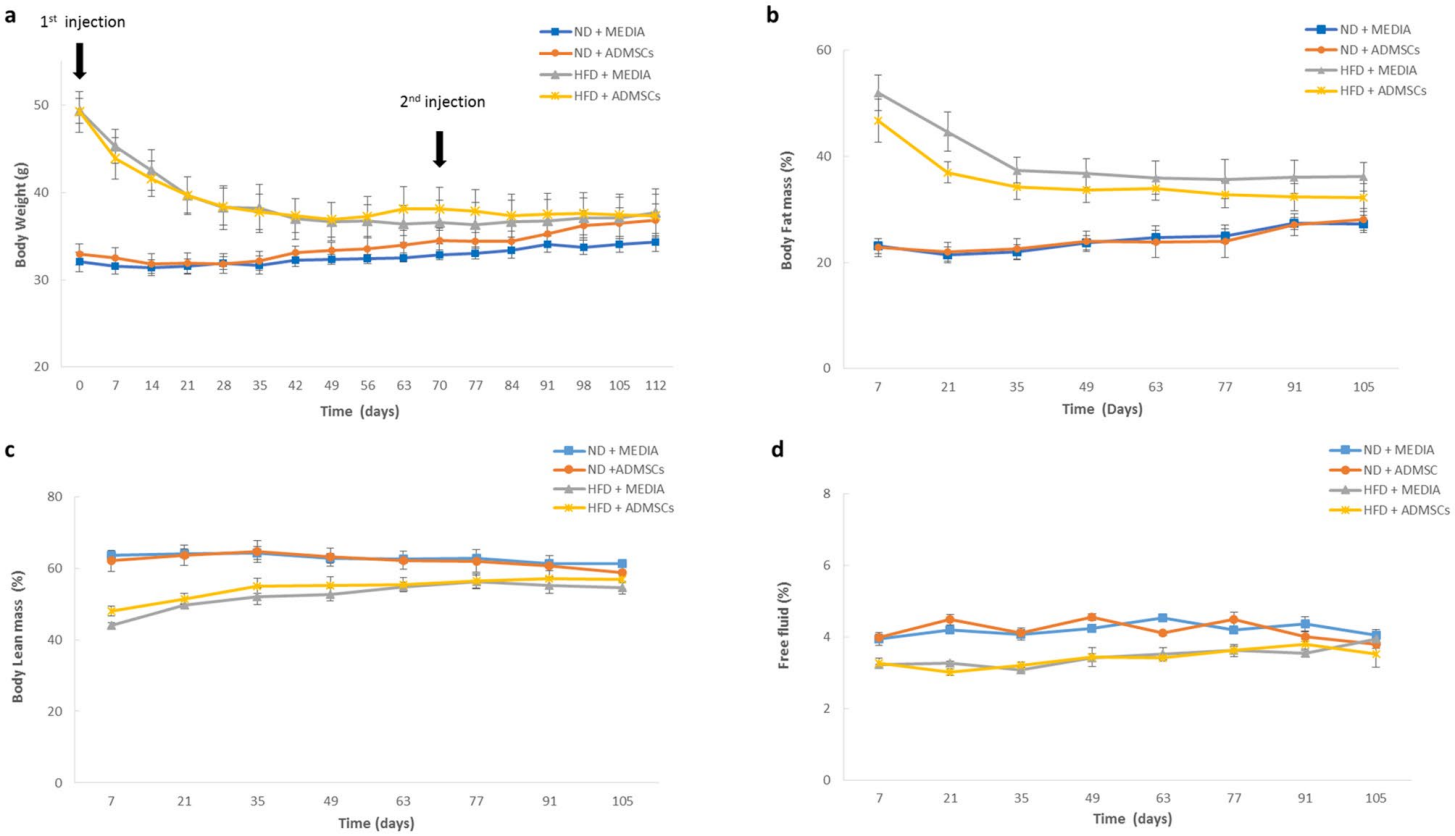
Changes in body weight and composition after MSC administration. (**a**) Changes in body weights (**b**) body fat mass percentages (**c**) body lean mass percentages and (**d**) free fluid percentages in normal mice and obese mice injected intraperitoneally (IP) with MEDIA and AD-MSCs at day 0 (1st injection) and day 70 (2nd injection). n = 6.

We examined the impact of IP injection of AD-MSCs on obesity and related comorbidities. After chronic HFD feeding (15 weeks), six mice of DIO and CHOW groups were injected with 4.2 × 10^7^ AD-MSCs/kg, while the control groups were injected with DMEM/F12 medium. A second dose followed after 10 weeks. After 15 weeks of feeding at baseline (day 0), the body weights of both DIO groups, HFD + MEDIA and HFD + ADMSCs, were significantly high (*p* < 0.001) and reached around 49.35 ± 1.42 g and 49.28 ± 2.3 g, respectively, as compared with the control groups, ND + media and ND + ADMSCs. However, at day 112, the body weights of both DIO groups (HFD + MEDIA and HFD + ADMSCs) decreased and reached 37.75 ± 2.6 g and 37.36 ± 2.47 g, but there were neither significant differences between HFD + MEDIA and ND + MEDIA groups, nor between HFD + ADMSCs and ND + ADMSCs groups (Fig. 3a).

Body composition analysis, a method used to measure the percentages of fat mass, lean mass and free fluids, was performed. DIO groups had significantly (*p* < 0.001) higher percentages of fat mass (52% and 46.7%) after chronic HFD feeding (day 7) as compared to controls (23%); however, after AD-MSCs treatments, the percentages of fat mass decreased significantly in HFD group from 46.73 ± 4.02% to 32.27 ± 2.59% in a trend similar to that of HFD + MEDIA group (Fig. 3b). On the other hand, the lean mass % (Fig. 3c) and fluid content % (Fig. 3d) were significantly lower in HFD-fed mice compared to CHOW-fed controls at baseline. Fifteen weeks after the first AD-MSCs administration, no significant changes were observed in both parameters in HFD-fed mice compared to controls.

### Effect of AD-MSCs on blood glucose levels, HbA1c and GTT

**Figure 4:**
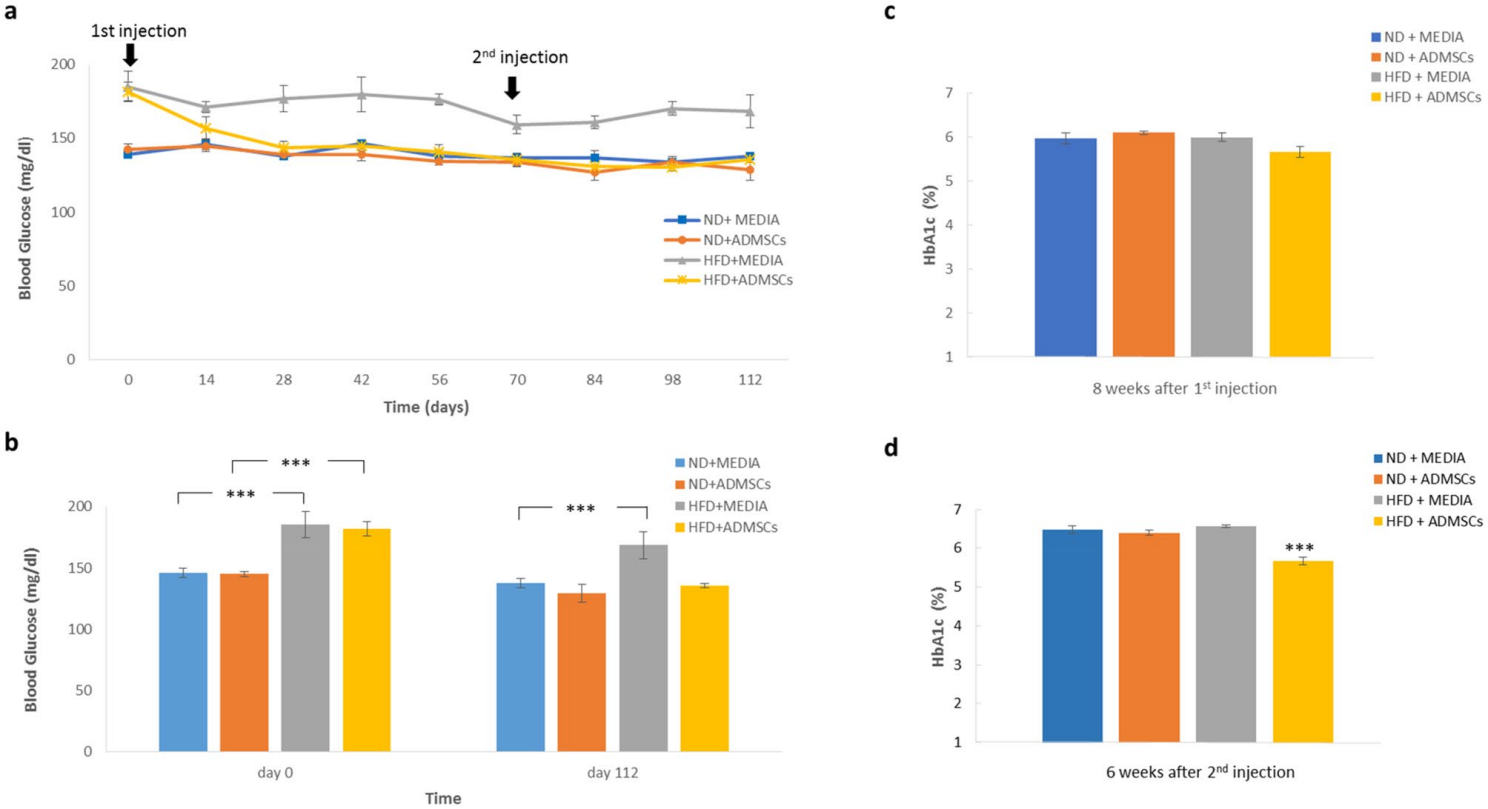
Blood glucose and glycated hemoglobin (HbA1c) levels after MSC administration. (**a**) and (**b**) Changes in blood glucose levels in ND- and HFD- fed mice injected IP twice with MEDIA and AD-MSCs, n = 6. (**c**) HbA1c levels at 8 weeks after 1st MSC injection and (**d**) 6 weeks after 2nd MSC injection in ND- and HFD- fed mice. n = 6, ***p < 0.001.

At baseline (day 0), HFD + MEDIA and HFD + ADMSCS groups showed significantly (*p* < 0.001) elevated blood glucose levels reaching 185.25 ± 10.4 mg/dl and 181.8 ± 6.15 mg/dl, respectively (Fig. 4a,b). Blood glucose levels at the end of the 16-week experimental period significantly decreased to 135.4 ± 1.63 mg/dl in the DIO mice treated with AD-MSCs as compared to those treated with media (168.5 ± 11.2 mg/dl). Normal groups (ND + MEDIA and ND + ADMSCs) showed normal glucose levels (Fig. 4a,b). Furthermore, HbA1c levels performed 10 weeks after first intervention, measured 6 ± 0.1% in HFD + MEDIA group as compared to 5.6 ± 0.12% in HFD + ADMSCs, but this was not significant (Fig. 4c). However, HbA1c levels at 6 weeks after second injection were significantly decreased (*p* < 0.001) in HFD-fed mice treated with AD-MSCs reaching 5.6 ± 0.1% (Fig. 4d).

**Figure 5:**
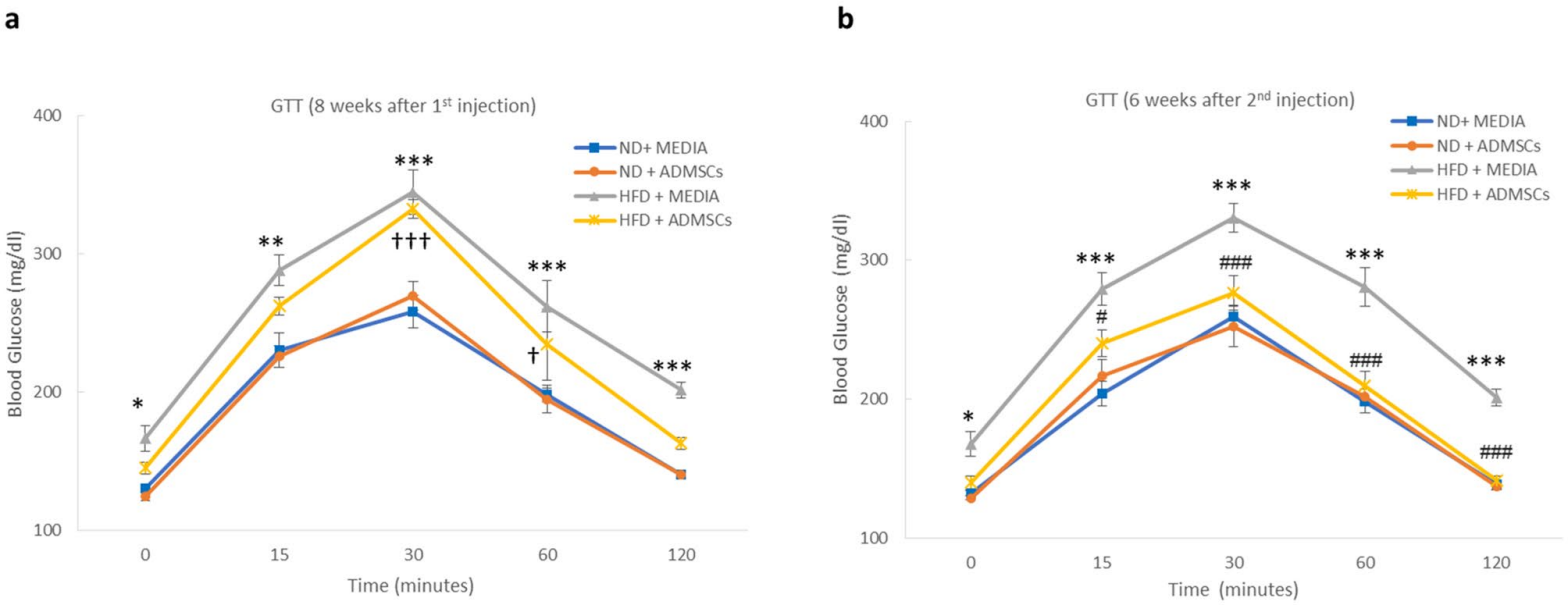
Effect of AD-MSC administration on Intraperitoneal Glucose Tolerance test (IPGTT). IPGTT was performed at (**a**) 8 weeks after 1st MSC injection and (**b**) 6 weeks after 2nd MSC injection in ND- and HFD- fed mice, n = 6. *Indicates statistical difference between HFD + MEDIA group and ND groups; ^†^Indicates statistical significance between HFD + ADMSC group and ND groups; ^#^Indicates statistical significance between HFD + ADMSC group and HFD + MEDIA group. *p < 0.05, **p < 0.01, ***p < 0.001, ^†^*p* < 0.05, ^†††^*p* < 0.001, ^#^*p* < 0.05, ^###^*p* < 0.001.

In addition to blood glucose and HbA1c tests, IPGTT was performed 8 weeks after first injection. HFD + MEDIA group compared to control groups showed a significant increase (*p* < 0.001) in glucose level > 340 mg/dl after 30 min of glucose injection which dropped to around 201.75 ± 5.8 mg/dl after 2 h. However, HFD + ADMSCs group showed a significant difference (*p* < 0.001) in glucose levels compared to control groups with the peak occurring at 30 min with a mean of 332.6 ± 6.84 mg/dl and reaching 163 ± 4.02 mg/dl at 2 h (Fig. 5a). 6 weeks after second injection, no significant differences in glucose levels among HFD + ADMSCs and control groups were observed (Fig. 5b). As a conclusion, AD-MSCs provided a positive effect on glycemic status and enhanced glucose disposal.

### Atherogenic index of plasma (AIP) levels

**Figure 6:**
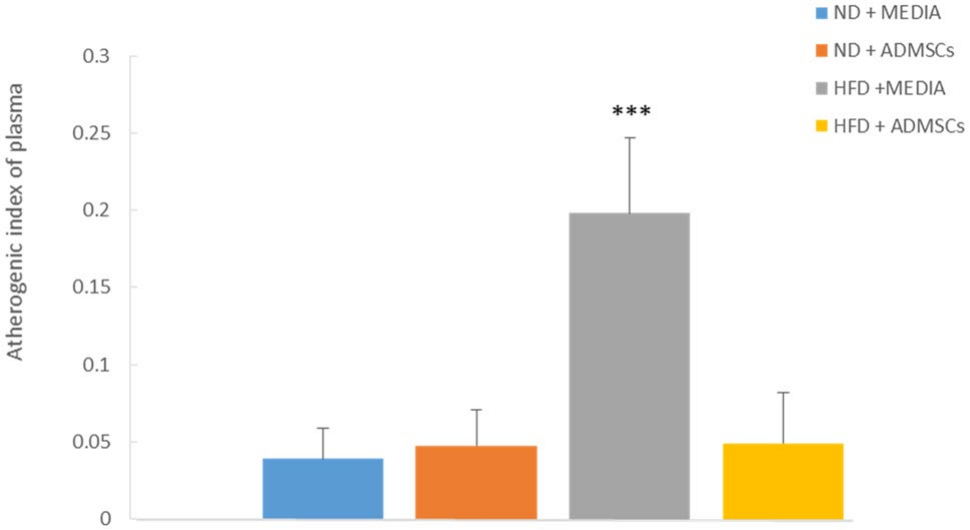
MSC treatment reduces the atherogenic index of plasma (AIP) in obese mice. AIP was measured at the end of the study in ND- and HFD- fed mice treated and untreated with AD-MSCs. n = 6, ***p < 0.001.

AIP is a very strong marker of cardiovascular diseases^15^. The effect of AD-MSCs on AIP was assessed by measuring HDL and TG values at the end of the study. Cardiovascular risk or AIP was the highest in HFD + MEDIA group as shown in Fig. 6. However, treatment of HFD group with AD-MSCs was sufficient to significantly reduce AIP to levels similar to those of normal diet groups.

### Evaluation of TNF-α and IL-6 levels

**Figure 7:**
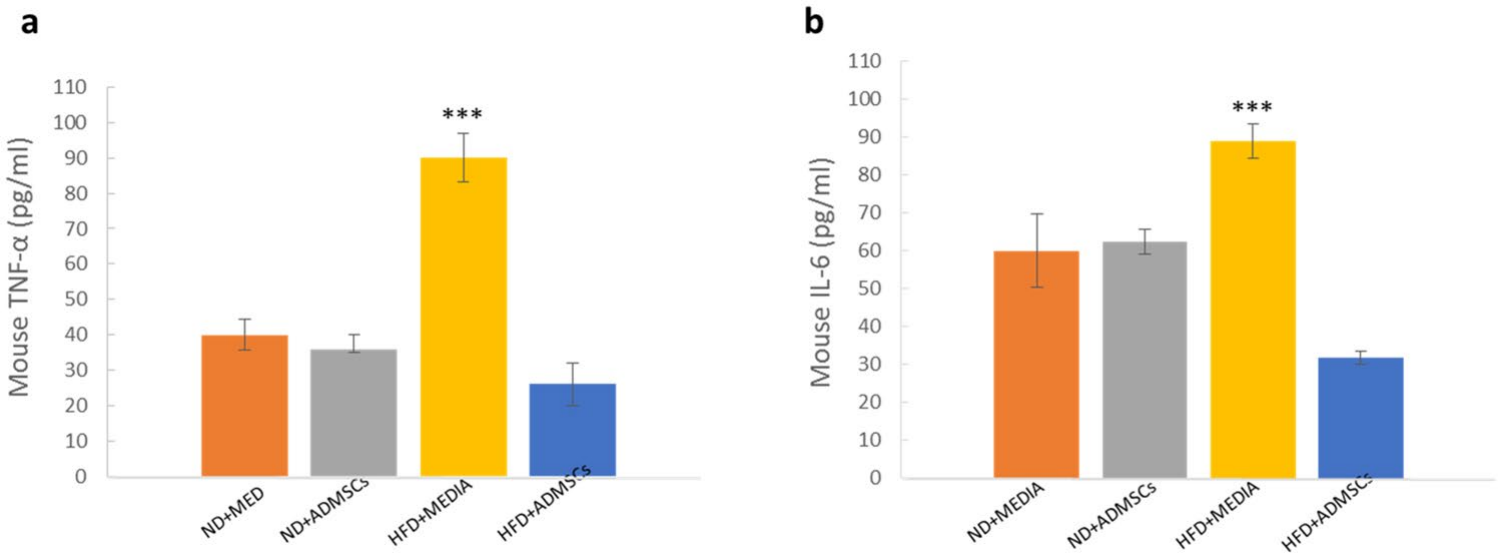
MSC treatment reduces pro-inflammatory cytokine levels in obese mice**.** (**a**) TNF-α and (**b**) IL-6 levels were measured by ELISA at the end of the study in ND- and HFD- fed mice treated and untreated with AD-MSCs. n = 6, ***p < 0.001.

Pro-inflammatory cytokines, TNF-α and IL-6, were measured at the end of the experiment. High levels of TNF-α and IL-6 were detected in both HFD and HFD + MEDIA groups; however, AD-MSCs transplantation caused a significant decrease in the protein levels (Fig. 7). Control groups (ND + MEDIA and ND + ADMSCs) showed normal levels of both cytokines.

## Discussion

Obesity is a serious public health concern that increases the chances of developing numerous diseases such as diabetes, cardiovascular diseases and other comorbidities^4^. Throughout the past half century, scientific progression has allowed management of obesity and its associated diseases via several measures^16^. However, all these management options are not without limitations; thus, there is an urgent need for new interventions. Stem cell therapy seems to be promising as an alternative strategy to manage obesity and its related problems^17,18^. As a matter of fact, mesenchymal stem cells have become attractive candidates due to the vital role they play in adipogenesis and hence have been proposed as a novel therapeutic option^19^. The differentiation potential of MSCs, relative ease of isolation and expansion coupled with their immunomodulatory, anti-inflammatory and homing properties have made MSCs extensively studied both in vitro and in vivo for the treatment of many diseases^17^. The study presented here was undertaken to determine the effect of AD-MSCs intervention on body weight, body composition, hyperglycemia, glucose tolerance and cardiovascular risk in high fat diet-induced obese mouse model as well as its mechanism of action.

In our work, after 15-week chronic feeding period, DIO mice demonstrated substantial increase in body weight with a weight gain of 110%. This was associated with significantly elevated fat mass (27.9% increase) and allevaited lean mass and free fluids. In contrast, mice fed with the CHOW diet remained healthy throughout the study. Herein, it was essential to inquire whether weight gained on a HFD was related to an increase in energy intake as a result of hyperphagia. Therefore, we measured the food intake in experimental mice over 24 h. No significant change in food intake was observed in DIO and control groups. Nevertheless, other studies like the one presented by Licholai et al. concluded that HFD induces an overconsumption^20^. In parallel, DIO mice gradually developed hyperglycemia with blood glucose levels exceeding 187 mg/dl and impaired glucose tolerance at the end of experimental feeding; whereas, control mice remained lean and normoglycemic with no metabolic abnormalities. This is consistent with previous studies^21–23^.

To test our hypothesis, we examined the impact of IP injection of human AD-MSCs on body weight and composition in both DIO mice and their controls. One of the strengths of our study is the utilization of human cells rather than murine MSCs in animal disease model, as this will more closely mimic the human milieu and help accelerate the translation of stem cell therapy to clinical practice. A previous review demonstrated the effectiveness of human MSCs administered across several different cross‐species models in 88 (93.6%) out of 94 experimental studies^24^.

The body weight of both DIO groups (HFD + ADMSC and HFD + MEDIA) decreased significantly with time to reach weight similar to the normal diet groups after 16 weeks (112 days) of treatment. Despite no change in overall body weight at the end of the treatment period, stem cell therapy was sufficient to reduce body fat mass in DIO mice with slight increase in lean mass and free fluids. This was similar to other studies showing that administration of MSCs did not affect body weight in DIO animals^25^. However, it contradicts with others demonstrating that AD-MSCs can induce a decrease in body weight in HFD-fed mice^26,27^. Herein, we have also shown that AD-MSCs can ameliorate hyperglycemia induced by the HFD and improve glucose tolerance and glucosylated Hb. In 2019, a study conducted by Shree et al. concluded that in obese/diabetic mice, AD-MSCs effectively decreased blood glucose levels and improved its tolerance in the experimental subjects^28^. It is well known that obesity is associated with increased risk of developing CVD. Therefore, to determine the effect of AD-MSC implantation on CVD risk in DIO mice, we measured AIP. AIP is a valuable novel marker to predict the risk of developing dyslipidemia and associated diseases such as cardiovascular diseases^15^. Reduced levels of AIP in HFD group treated with AD-MSCs showed the beneficial aspect and the possible anti-atherosclerotic effects of AD-MSCs. Our study agreed with a number of previous studies showing an improvement in serum lipid profile after AD-MSCs transplantation^2,14,28^. Moreover, when measuring inflammatory cytokines, it was striking that AD-MSCs treatment decreased TNF-α and IL-6 serum levels to concentrations similar to that of ND groups. This could explain the mechanism by which MSCs exert their hypoglycemic and cardioprotective effects by suppressing inflammatory markers, TNF-α and IL-6, that both play a role in the progression of diabetes and atherosclerosis^29,30^. Similar to our work, other laboratories have shown attenuation in inflammatory markers upon administration of MSCs in HFD-fed animal models^31,32^.

It is critically important to understand that obesity is associated with macrophage accumulation in adipose tissue^33,34^. It has been reported that diet‐induced obesity activates adipose tissue macrophages (ATM) into pro‐inflammatory M1 macrophages that produce a variety of pro-inflammatory cytokines such as TNF‐α and IL‐6, which contribute to the development of diabetes and atherosclerosis^35,36^. In our study, in line with previous studies, MSC administration lowered the expression of pro‐inflammatory cytokines^37,38^. Owing to their immunomodulatory and anti-inflammatory properties, we postulate that MSC infusion produced significant anti-diabetic effects via soluble factors in part through directing ATM into anti-inflammatory M2 state and subsequently suppressing the secretion of pro-inflammatory mediators, thus ameliorating the inflammatory microenvironment^32,39^.

In conclusion, our work showed effectiveness in treating obesity-associated diabetes as well as protective effect against CVD as shown by the AIP, which might be partly due to the attenuation of inflammatory mediators, TNF-α and IL-6. Despite the importance of our work in proving the functionality of using AD-MSCs for treating obesity-related diseases in an animal model, more future experiments should be conducted before moving forward into assessing AD-MSCs  transplantation in treating obesity-concomitant diseases in humans.

## Methods

### Animals and diets

Twenty-four male C57BL/6 mice aged 8 weeks (25–28 g) were hosted in a controlled environment at the Animal Care Facility at the American University of Beirut with free access to water and food and a 12-h light/dark cycle with a temperature of 22 ± 2 °C. A period of seven days was used as an adaptation period before conduction of the experimentation. All experimental procedures were performed in agreement with the NIH Guidelines for the Use of Animals in Research and approved (18-12-510) by the Institutional Animal Care and Use Committee (IACUC) at the American University of Beirut. Our study was also carried out in accordance with the ARRIVE (Animal Research: Reporting of In Vivo Experiments) guidelines^40^. Mice were then randomly divided into two groups. The normal diet (ND) group was fed a normal CHOW diet with 10% of total calories from fat and the other group fed a HFD (Research Diets, Inc, D12492, New Jersey, USA) with 60% of calories from fat for a period of 15 weeks to generate the high fat diet-induced obese (DIO) mouse model.

### Adipose-derived mesenchymal stem cells (AD-MSCs) culture

Human AD-MSCs (a kind gift from Reviva Regenerative Medicine Center, Bsalim, Lebanon) were maintained in a DMEM/F12 medium with 10% FBS and 1% antibiotic–antimycotic at 37ºC and 5% CO_2_ atmosphere. The medium was changed every 48 h and cells were split when they reached 80–90% confluence. At passage 4, cells were trypsinized and resuspended in DMEM/F12 media ready for animal treatment.

### Experimental design

**Figure 8:**
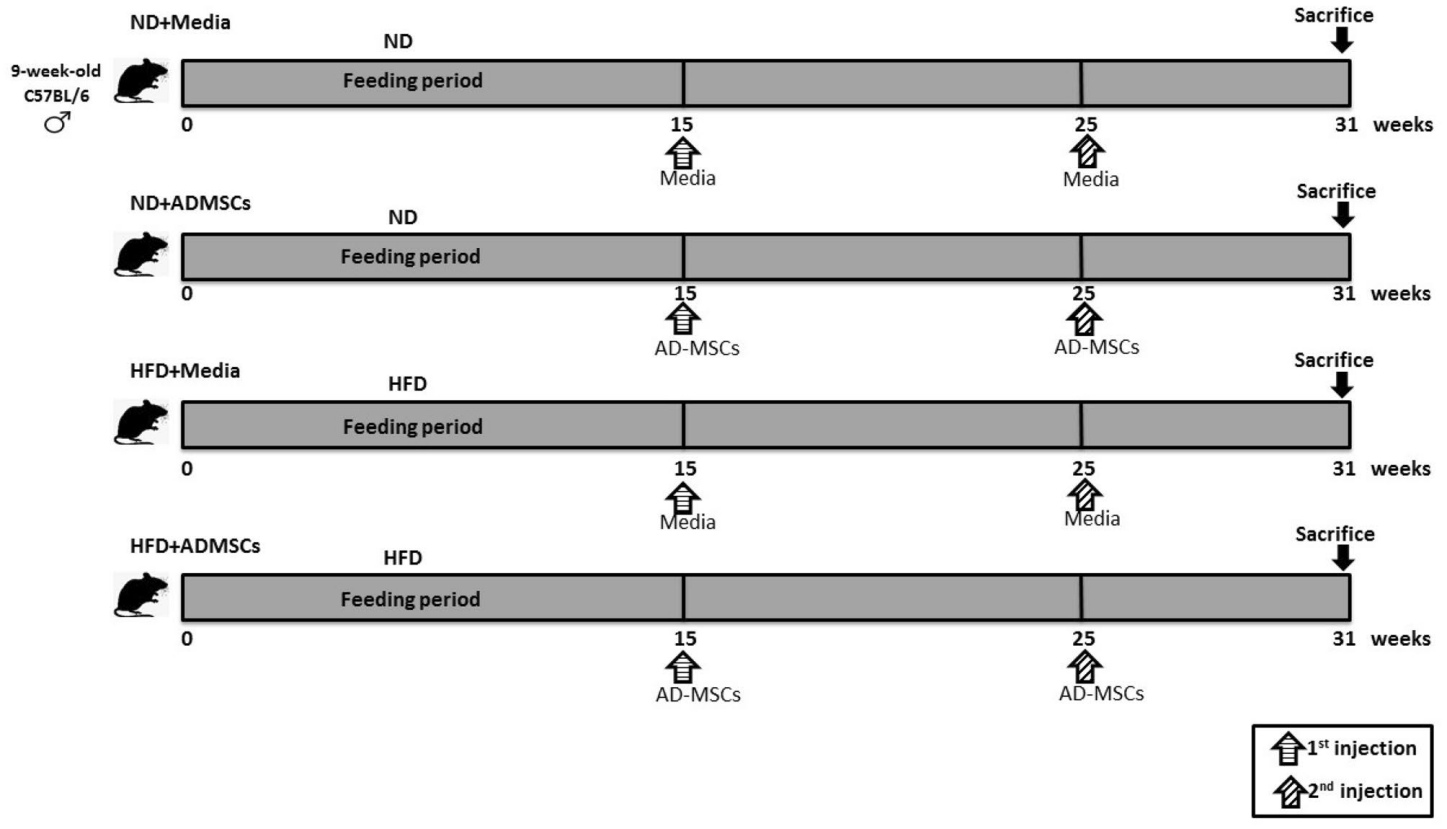
Schematic diagram of the experimental design of the study. Male C57BL/6 mice (n = 24) were fed with normal diet (ND) or (HFD) for 15 weeks. Following the feeding period, ND- and HFD-fed mice were returned to standard diet, then randomly divided into 2 groups each (n = 6). One group received 2 doses of adipose-derived mesenchymal stem cells (AD-MSCs); while the other received the media to serve as control.

After 15 weeks of chronic feeding with the HFD or CHOW diet, the HFD was removed and half of the animals in each group (n = 6) were injected intraperitoneally (IP) with 4.2 × 10^7^ cells/kg AD-MSCs suspended in media (ND + ADMSCs and HFD + ADMSCs groups) and the other halves (n = 6) were injected with DMEM/F12 media as controls and labelled ND + MEDIA or HFD + MEDIA. A second treatment (4.2 × 10^7^ cells/kg) was repeated 10 weeks after the first injection. Similarly, control groups were injected with an equal volume of DMEM/F12 medium. The experimental procedure design is summarized in Fig. 8. All animals were sacrificed 6 weeks after the second injection and serum collected. For serum collection, blood was withdrawn from the retro-orbital sinus, centrifuged at 12 g for 15 min at 4 °C, then serum stored at − 20 °C for further analysis.

### Monitoring food intake, body weight and body composition

Mouse food intake was monitored before the first treatment injection at week 12 during a 24-h period. Body weights were also measured once per week and body composition analysis was performed using the Minispec LF110 Live Mice Analyzer (Bruker, USA) every 2 weeks for all mice. Briefly, the animal without anaesthesia was guided into a red restrainer to reduce the animal’s anxiety. A tight-fitting plunger was also inserted into the restariner to maintain the mouse immobile during the duration of the scan, which is around 2 min. The fat mass, lean mass, and free body fluid were measured before releasing the animal from the restrainer.

### Measuring blood glucose, glycated hemoglobin and glucose tolerance

Blood glucose levels were measured every 2 weeks from a drop of blood by tail incision using Accu-Chek blood glucose meter and Accu-Chek Active glucose strips (ROCHE, Switzerland). Whereas, glycated hemoglobin (HbA1c) was assessed using the BIOHRMES HbA1c Analyzer (BIOHERMES, USA) at the 8th week following the first injection, and the 6th week following the second injection. Besides, the intraperitoneal glucose tolerance test (IPGTT) was performed using the Accu-Check blood glucose meter. Briefly, after six hours of fasting, mice body weights and fasting blood glucose levels were recorded (t = 0 min). Mice were then injected with 20% glucose. The volume of the glucose injection was calculated according to the formula: volume = 10 × lean body mass (g)^41^. Blood glucose levels were then measured at 15, 30, 60 and 120 min post-injection.

### Determination of AIP levels

Triglycerides (TG) and high-density lipoprotein (HDL) cholesterol levels were measured at the end of the experiment using the Mission Cholesterol Monitoring System (ACON Labs Inc., USA) to determine the atherogenic index of plasma (AIP) that was calculated as AIP = Log[TG/HDL]^15^.

### Serum TNF-α and IL-6 protein levels

Tumor necrosis factor-α (TNF-α) and interleukin-6 (IL-6) concentrations in the serum were assayed using the mouse TNF-α (ab208348) and IL-6 (ab222503) SimpleStep ELISA kits (Abcam) according to the manufacturer's instructions. The absorbance was measured spectrophotometrically using a Thermo Scientific Multiskan FC ELISA microplate reader at 450 nm, and the cytokine concentrations were determined based on the optical densities obtained from the standards.

### Statistical analysis

Data were analysed using the statistical package for social sciences (SPSS 24 for Windows) and presented as mean ± SEM. A two-way analysis of variance (ANOVA) test was performed to assess the effects of the two variables (age, treatments) and their interactions on the body weight, blood glucose level and body composition (body fat mass, body lean mass, free fluid) of mice. This was followed by testing for statistically significant difference between variables using Bonferroni post-hoc test. *P* value < 0.05 was considered to be statistically significant.
