# Author Correction: The therapeutic effects of adipose-derived mesenchymal stem cells on obesity and its associated diseases in diet-induced obese mice

**DOI:** 10.1038/s41598-021-91860-6

**Published:** 2021-06-08

**Authors:** Hala jaber, Khodr Issa, Ali Eid, Fatima A. Saleh

**Affiliations:** 1 Department of Nutrition and Dietetics, Faculty of Health Sciences, Beirut Arab University, Beirut, Lebanon; 2 Department of Molecular Diagnostics, Doctors’ Center Laboratories, Beirut, Lebanon; 3 Department of Pharmacology and Toxicology, Faculty of Medicine, American University of Beirut, Beirut, Lebanon; 4 Department of Basic Medical Sciences, College of Medicine, QU Health, Qatar University, Doha, Qatar; 5 Biomedical and Pharmaceutical Research Unit, QU Health, Qatar University, Doha, Qatar; 6 Department of Medical Laboratory Technology, Faculty of Health Sciences, Beirut Arab University, Beirut, 115020 Lebanon

Correction to: *Scientific Reports* 10.1038/s41598-021-85917-9, published online 18 March 2021

The original version of this Article omitted the Acknowledgements and Funding section. The Acknowledgements section now reads:

“The authors thank Dr. Samer Kharroubi from American University of Beirut for statistical guidance and Dr. Charbel Khalil from Reviva Regenerative Medicine Center, Lebanon, for the kind gift of AD-MSCs.”

The Funding section now reads:

“This study was funded by Beirut Arab University (BAU) and the Lebanese National Council for Scientific Research (CNRS-L).”

The original Article has been corrected.

